# Buffalo Milk Whey Activates Necroptosis and Apoptosis in a Xenograft Model of Colorectal Cancer

**DOI:** 10.3390/ijms23158464

**Published:** 2022-07-30

**Authors:** Nunzio Antonio Cacciola, Angela Salzano, Nunzia D’Onofrio, Tommaso Venneri, Paola De Cicco, Francesco Vinale, Orsolina Petillo, Manuela Martano, Paola Maiolino, Gianluca Neglia, Ciro Campanile, Lorella Severino, Carmine Merola, Francesca Borrelli, Maria Luisa Balestrieri, Giuseppe Campanile

**Affiliations:** 1 Department of Veterinary Medicine and Animal Production, University of Naples Federico II, Via F. Delpino 1, 80137 Naples, Italy; nunzioantonio.cacciola@unina.it (N.A.C.); angela.salzano@unina.it (A.S.); paola.decicco@unina.it (P.D.C.); frvinale@unina.it (F.V.); manuela.martano@unina.it (M.M.); paola.maiolino@unina.it (P.M.); gianluca.neglia@unina.it (G.N.); lseverin@unina.it (L.S.); giucampa@unina.it (G.C.); 2 Research Institute on Terrestrial Ecosystems (IRET), UOS Naples-Consiglio Nazionale delle Ricerche (CNR), Via Pietro Castellino 111, 80131 Naples, Italy; orsolina.petillo@cnr.it; 3 Department of Precision Medicine, University of Campania Luigi Vanvitelli, Via L. De Crecchio 7, 80138 Naples, Italy; nunzia.donofrio@unicampania.it (N.D.); marialuisa.balestrieri@unicampania.it (M.L.B.); 4 Department of Pharmacy, School of Medicine and Surgery, University of Naples Federico II, 80131 Naples, Italy; tommaso.venneri@unina.it; 5 Institute of Genetics and Biophysics “Adriano Buzzati Traverso”, Consiglio Nazionale delle Ricerche (CNR), 80131 Naples, Italy; ciro.campanile@igb.cnr.it; 6 Faculty of Bioscience and Technology for Food, Agriculture and Environment, University of Teramo, Via Balzarini 1, 64100 Teramo, Italy; cmerola@unite.it

**Keywords:** delactosed milk whey, necroptosis, apoptosis, xenograft, colorectal cancer

## Abstract

Recent pharmacological research on milk whey, a byproduct of the dairy industry, has identified several therapeutic properties that could be exploited in modern medicine. In the present study, we investigated the anticancer effects of whey from Mediterranean buffalo (*Bubalus bubalis*) milk. The antitumour effect of delactosed milk whey (DMW) was evaluated using the HCT116 xenograft mouse model of colorectal cancer (CRC). There were no discernible differences in tumour growth between treated and untreated groups. Nevertheless, haematoxylin and eosin staining of the xenograft tissues showed clearer signs of different cell death in DMW-treated mice compared to vehicle-treated mice. Detailed biochemical and molecular biological analyses revealed that DMW was able to downregulate the protein expression levels of c-myc, phospho-Histone H3 (ser 10) and p-ERK. Moreover, DMW also activated RIPK1, RIPK3, and MLKL axis in tumour tissues from xenograft mice, thus, suggesting a necroptotic effect. The necroptotic pathway was accompanied by activation of the apoptotic pathway as revealed by increased expression of both cleaved caspase-3 and PARP-1. At the molecular level, DMW-induced cell death was also associated with (i) upregulation of SIRT3, SIRT6, and PPAR-γ and (ii) downregulation of LDHA and PPAR-α. Overall, our results unveil the potential of whey as a source of biomolecules of food origin in the clinical setting of novel strategies for the treatment of CRC.

## 1. Introduction

Colorectal cancer (CRC) is the third most commonly diagnosed cancer in women and men worldwide, with prevalence increasing even in traditionally low-risk countries [1]. Nevertheless, improvements in CRC treatment strategies, lower risk factors prevalence, and preventive CRC screening have led to both lower mortality and incidence of CRC [1]. Similarly, CRC is also a serious problem in pets [2,3]. Recently, it has been demonstrated that canine CRC occurs spontaneously and has a similar clinical presentation and pathophysiology to human cancers [3]. Furthermore, they live in the same environment as humans and are therefore exposed to the same carcinogens [3]. Surgery and chemotherapy have long been recognised as the first treatment option for CRC patients [4,5]. However, the occurrence of serious side effects associated with chemotherapy encourages researchers to search for new bioactive molecules from different sources to develop new antitumour drugs that could overcome these side effects and be useful for both humans and pets.

The agri-food industry produces a large amount of waste and residues along the entire supply chain that still contain a considerable number of bioactive compounds such as polyphenols (phenolic acids, flavonoids, proanthocyanidins, anthocyanins), saponins, tannins, alkaloids, sterols, triterpenes, peptides, and carbohydrates [6,7]. In recent decades, several studies have widely shown that these compounds possess various biological activities, including antioxidant, antibacterial, antifungal, antiviral, antimicrobial, antidiabetic, anticancer, antidiarrheal, antihypertensive, antimutagenic, and anti-inflammatory properties [8,9].

The dairy industry occupies an important position among food industries due to its advanced technology and widely used products such as cheese, yoghurt, butter, cream, and ice cream [10,11]. In addition to milk, the residual whey obtained after ricotta production contains significant amounts of betaines, L-carnitine, and short-chain acylcarnitines [12]. In recent years, experimental research has revealed the pharmacological potential of dairy whey in improving energy balance and muscle mass in cancer patients [13,14]. The potential anticancer effect of a 3 kDa fraction of whey from Italian Mediterranean buffalo (*Bubalus bubalis*) milk has been demonstrated in in vitro CRC cell models [12]. So far, however, the anticancer effect of whey has not been demonstrated in preclinical in vivo studies.

In the present study, we investigated the effect of a delactosed milk whey (DMW) from Italian Mediterranean buffalo on tumour progression in vivo and the molecular players of the anticancer mechanisms in colon cancer mouse xenograft tissues.

## 2. Results

### 2.1. Milk Whey Metabolome Profiling

The major constituents identified in DMW are shown in Table 1.

### 2.2. DMW Does Not Significantly Reduce Tumour Growth in Xenograft Mice

**Figure 1:**
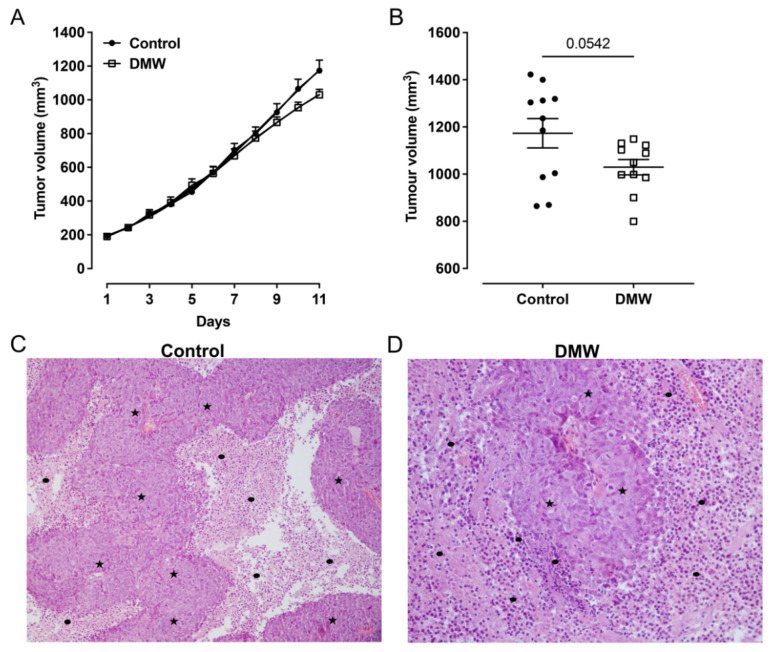
DMW does not significantly reduce CRC growth in vivo. (**A**,**B**) Effect of DMW (0.2 mL/mouse, given by oral gavage) on in vivo tumour growth, measured as tumour volume (mm^3^), over an 11-day period. Each point is the mean ± SEM of 11 mice. (**C**,**D**) Histological features of control- and DMW-treated samples. Representative images of (**C**) control sample showed adenocarcinoma (stars) with central necrosis (dots) (H-E; original magnification ×10) and (**D**) DMW-treated sample showed adenocarcinoma (stars) with large areas of necrosis, which surround and invade neoplastic proliferation (dots) (H-E; original magnification ×40).

**Figure 2:**
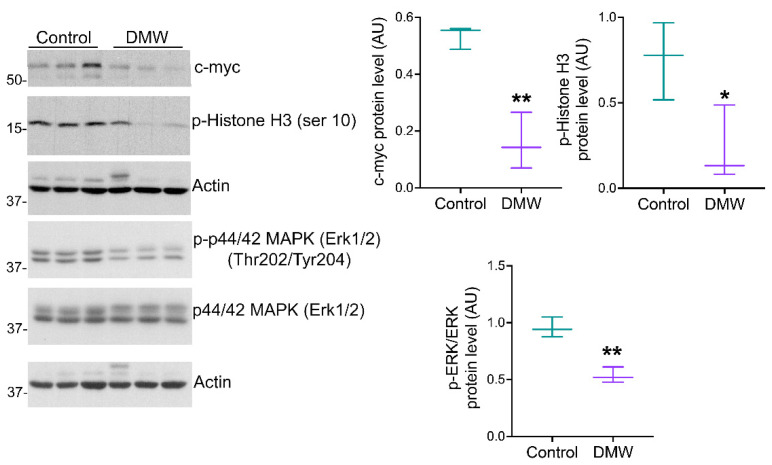
DMW downregulates the protein expression levels of proliferative markers. Western blot analysis showing the protein expression levels of c-myc, phospho-Histone H3 (ser 10), phospho-ERK, and ERK in xenograft tissue of DMW-treated mice and vehicle-treated mice. The right side of the panel shows the densitometry analysis of the above proteins. Signals were normalised on the housekeeping protein actin. Data represent the mean ± SEM of three tissues from vehicle- or DMW-treated mice. * *p* < 0.05, and ** *p* < 0.01 vs. control (vehicle-treated mice).

To determine whether DMW affected tumour development in mice, a human colon cancer xenograft model was created using athymic mice. Human colon cancer cells, HCT116 cells, were transplanted into the back of the athymic mice to induce tumour development before DMW or vehicle treatment. A slight reduction in tumour volume was observed in DMW-treated mice at day 10 and 11, although it did not reach statistical significance (Figure 1A). The same trend was observed in the postmortem excited tumours of the DMW-treated mice compared to vehicle-treated mice (*p* < 0.063) (Figure 1B). Next, we investigated the effect of DMW on xenograft tissues from animals treated with or without DMW. All control samples showed proliferation of neoplastic cells with central necrosis (Figure 1C), which was mild, moderate, and severe in 7/11, 3/11, and 1/11 samples, respectively (Supplementary Table S1). In all DMW-treated samples (11/11), proliferation of neoplastic cells with large areas of infarct-like necrosis was found (Figure 1D). These areas surrounded the tumour and tended to invade it (Supplementary Figure S1A). The neoplastic cells showed clear signs of pyknosis, karyorrhexis, and karyolysis, which was confirmed by the presence of numerous ghost cells (Supplementary Figure S1B). Necrosis was mild in 3/11 samples, moderate in 3/11, and severe in 5/11 samples (Supplementary Table S1). Furthermore, using Western blot analysis, we demonstrated that DMW was able to cause downregulation of several markers involved in cell proliferation. As shown in Figure 2, DMW significantly decreased the protein expression of c-myc, phospho-histone H3, and p-ERK in xenografts from DMW-treated mice, demonstrating an antiproliferative effect.

### 2.3. DMW Triggers a Pro-Inflammatory Milieu and Induces Necroptosis

**Figure 3:**
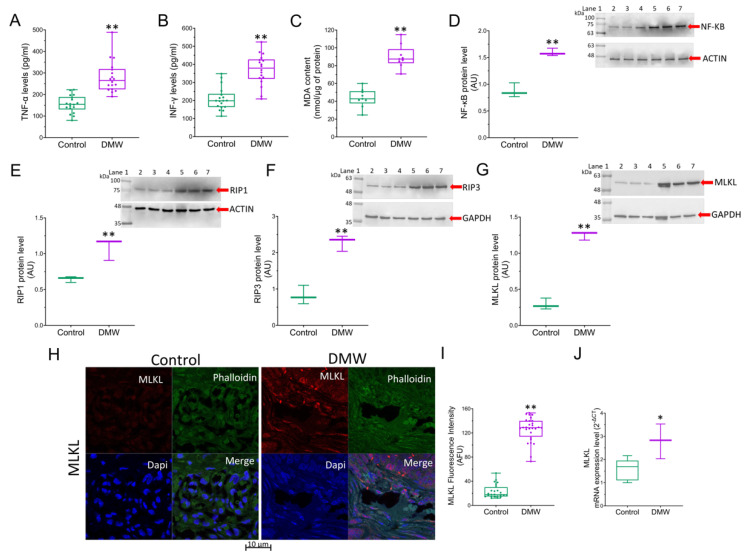
DMW activates inflammation and necroptosis in xenograft model of colorectal cancer. (**A**,**B**) Expression levels detected by ELISA assay of TNF-α and INF-γ, (**C**) MDA content and (**D**–**G**) immunoblotting analysis of NF-κB, RIP1, RIP3, and MLKL in control and DMW-treated mice. (**H**–**J**) Representative immunofluorescence images, analysis, and mRNA expression of MLKL in control and DMW-treated mice. Fluorescence intensity determination, reported as boxplots representing the densitometric mean values of arbitrary fluorescence units (AFU). For immunoblotting study, boxplots represent the densitometric mean values, expressed as arbitrary units (AU) of n = 5 different experiments. Protein expression was determined after normalisation with α-tubulin or GAPDH as internal control, with ImageJ software. Lane 1 = molecular marker; Lanes 2, 3, and 4 = control mice; Lanes 5, 6, and 7 = DMW-treated mice. * *p* < 0.05 vs. control mice; ** *p* < 0.01 vs. control mice.

The observation of dying cells that exhibited a “ghostly” morphology resembling necrosis prompted us to investigate the expression of the necroptotic pathway and consequently the expression of inflammatory markers in the xenograft tissues of mice treated with vehicle or DMW. Results showed that the DMW ability in inducing upregulation of the pro-inflammatory cytokine levels (TNF-α and INF-γ)was significantly (*p* < 0.01) higher than the control (Figure 3A,B). Similarly, the MDA content and NF-κB protein expression levels were also significantly (*p* < 0.01) higher in the DMW than in the control (Figure 3C,D). These pro-inflammatory effects were accompanied by induction of necroptosis. Indeed, Western blot analysis showed a significant (*p* < 0.01) increase in the expression of both RIP1 and RIP3 proteins in DMW-treated xenograft mice compared to control mice (Figure 3E,F). Results obtained by immunoblotting, immunofluorescence, and qRT-PCR analyses also showed the DMW efficacy in positively modulating the protein (Figure 3G–I) and mRNA (Figure 3J) expression levels of MLKL, the terminal effector in necroptotic signalling.

### 2.4. DMW Induces Apoptosis in Xenograft Mice

**Figure 4:**
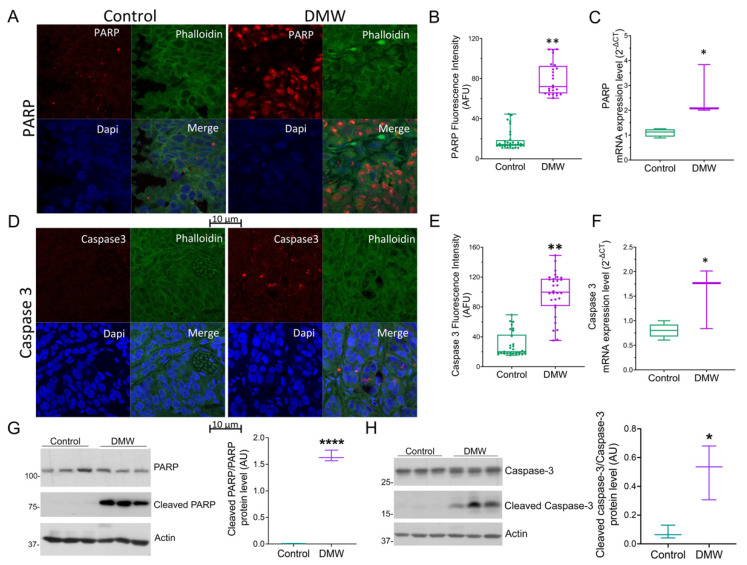
DMW promotes apoptosis activation in a xenograft model of colorectal cancer. (**A**–**C**) PARP and (**D**–**F**) caspase 3 protein and mRNA expression level determined by immunofluorescence and quantitative real-time PCR in control and DMW-treated mice. Fluorescence intensity determination, reported as boxplots representing the densitometric mean values of arbitrary fluorescence units (AFU). (**G**,**H**) Western blot analysis and densitometric analysis for PARP, cleaved PARP, caspase-3 and cleaved caspase-3 detection in tissues from control and DMW-treated mice. Signals were normalised as cleaved PARP/PARP and cleaved caspase-3/caspase-3 ratios. Data represent the mean ± SEM of three individual xenograft tissues. * *p* < 0.05, ** *p* < 0.01 and **** *p* < 0.0001 vs. control mice.

Since it is known that necroptosis and apoptosis are linked [15,16,17], we decided to evaluate the ability of DMW to activate apoptosis in xenograft tissues. Immunofluorescence and qRT-PCR analyses showed upregulation of PARP (Figure 4A–C) and procaspase-3 (Figure 4D–F) in mice treated with DMW compared to control mice. In detail, DMW treatment increased PARP and procaspase-3 protein and mRNA levels compared to control. The effect of DMW on cleaved caspase-3/caspase-3 and cleaved PARP/PARP ratio(s) was also examined using Western blot analysis. As shown in Figure 4G,H cleaved caspase-3/caspase-3 and cleaved PARP/PARP ratio(s) significantly increased in the xenograft tissues from DMW-treated mice compared to vehicle-treated tissues.

### 2.5. DMW Affects SIRT6 and SIRT3 Expression and Activity

**Figure 5:**
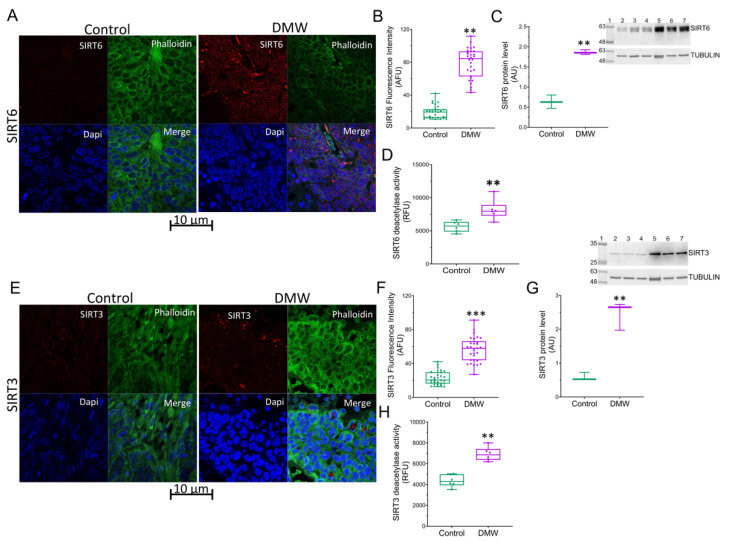
DMW modulates SIRT6 and SIRT3 expression and activity in xenograft model of colorectal cancer. Representative immunofluorescence images and analysis, protein expression levels and deacetylase enzymatic activity of (**A**–**D**) SIRT6 and (**E**–**H**) SIRT3 in control and DMW-treated mice. Fluorescence intensity determination, reported as boxplots representing the densitometric mean values of arbitrary fluorescence units (AFU). For immunoblotting study, boxplots represent the densitometric mean values, expressed as arbitrary units (AU) of n = 5 different experiments. Protein expression was determined after normalisation with α-tubulin as internal control with ImageJ software. Lane 1 = molecular marker; Lanes 2, 3, and 4 = control mice; Lanes 5, 6, and 7 = DMW-treated mice. ** *p* < 0.01 and *** *p* < 0.001 vs. control mice.

A previous study demonstrated the involvement of sirtuins in the in vitro antitumour effect of DMW [12]. To substantiate the results of previous in vitro studies and to bridge the gap between in vivo and in vitro research, we investigated the involvement of these proteins in the effects of DMW. A significant upregulation of SIRT6 protein levels were detected in tissues of xenograft mice treated with DMW (Figure 5A–C) compared to control mice and similar results (Figure 5D) were recorded for enzyme activity. Similar results were observed when assessing SIRT3 protein expression levels and enzyme activity (Figure 5E–H). 

### 2.6. DMW Triggers Modulation of the Metabolic Pathway

**Figure 6:**
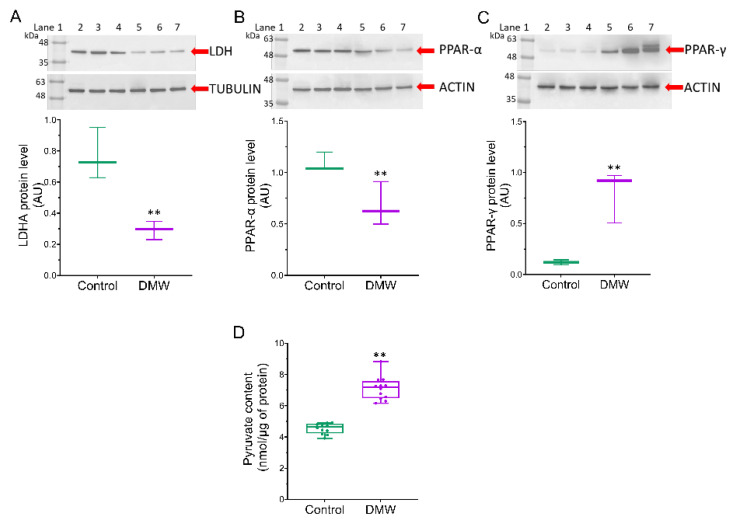
DMW effects on mitochondrial metabolic markers of xenograft model of colorectal cancer. (**A**–**C**), LDHA, PPAR-α, and PPAR-γ protein levels detected on control and DMW-treated mice. Protein expression was determined after normalisation with α-tubulin or actin as internal control, with ImageJ software and values expressed as arbitrary units (AU). Lane 1 = molecular marker; lanes 2, 3, and 4 = control mice; lanes 5, 6, and 7 = DMW-treated mice. (**D**), Pyruvate content in tissue from control and DMW-treated mice was expressed as nmol/µg of protein. ** *p* < 0.01 vs. control mice.

Since SIRT3 exerts its effect on tumourigenesis by affecting genes such as PPAR-α and PPAR-γ [12] and considering that lactate dehydrogenase A (LDHA) is overexpressed in CRC [18,19,20,21], we examined whether DMW affected the expression of these proteins. LDHA and PPAR-α protein expression levels were significantly higher in control mice compared to DMW-treated mice (Figure 6A,B). In contrast, higher PPAR-γ levels and pyruvate content were detected in the tissues of DMW-treated mice compared to control mice (Figure 6C,D).

## 3. Discussion

Despite appropriate prevention strategies that have reduced mortality rates, colorectal cancer (CRC) remains a serious, problematic human disease that requires the identification of new therapeutic treatments. Here, we investigated the antineoplastic effect of delactosed milk residual whey (DMW) after ricotta production in vivo using a xenograft model of CRC that provides insight into its potential action in CRC. We found that although 11 days of treatment with DMW did not significantly suppress the growth of CRC as measured by volume, it did induce necroptosis and apoptosis.

Indeed, haematoxylin and eosin staining in the xenograft tumour sections of DMW-treated mice showed severe signs of necrosis and cellular inflammatory infiltration compared to control mice. Since we were not allowed to continue the experiment once the tumours reached a size of 1000 mm^3^, we cannot exclude the possibility that the size of the xenograft tumours in DMW-treated mice will decrease even further if the experiment continued. Accordingly, we found that the DMW treatment reduced the protein expression levels of c-myc, phospho-histone H3, and p-ERK which are well-known markers of cell proliferation.

Necroptosis is usually considered a highly pro-inflammatory form of cell death, as it releases intracellular “danger signals” such as the pro-inflammatory TNF-α and IFN-γ [22,23,24]. Furthermore, necroptotic signalling is triggered by a variety of signals, including death receptor ligands, and controlled by receptor-interacting protein kinases 1 and 3 (RIPK1 and RIPK3) and mixed-lineage kinase domain-like pseudokinase (MLKL), which form a regulatory necrosome complex [25]. To determine whether DMW treatment induces necroptosis, we measured the levels of pro-inflammatory cytokines and protein expression of RIPK1, RIPK3, and MLKL in xenograft tissues from vehicle- and DMW-treated mice. Our results showed that DMW treatment (i) had potent and direct pro-inflammatory effects in xenograft tumour tissues, as reflected by increased expression of pro-inflammatory cytokines (e.g., TNF-α and IFN-γ) and activation of the NF-κB pathway, and (ii) caused a significant increase in RIPK1, RIPK3, and MLKL protein expression in the xenograft mice compared to vehicle-treated mice. The increase in MLKL expression levels was also confirmed by immunofluorescence and qRT-PCR analyses. All these data are suggestive of a DMW-induced activation of the necroptotic pathway.

It is well-established that the final step of necroptosis involves the induction of oxidative stress leading to the generation of reactive oxygen species, which in turn trigger the peroxidation of membrane lipids, resulting in the release of malondialdehyde (MDA), an oxidative marker altered in CRC [26,27]. Our results revealed that DMW increased MDA levels in DMW-treated xenograft mice compared to the control group (vehicle-treated mice), suggesting that probably a greater oxidative stress could occur in DMW-treated mice, thus supporting the necroptotic effect of DMW.

Apoptosis and necroptosis occur simultaneously in a variety of diseases in animals and humans [15,16,17]], suggesting that these cell death events are inter-related and not completely separate. In the present study, we also investigated the possible activation of the apoptotic pathway by DMW by examining the expression of caspase-3 and poly (ADP -ribose) polymerase 1 (PARP1, a known caspase substrate) by Western blotting, immunofluorescence, and qRT-PCR analyses. The results show an increase in protein and mRNA expression of caspase-3 and PARP1 in DMW-treated xenograft mice compared to mice treated with vehicle.

In our previous report, we documented an involvement of sirtuins, enzymes belonging to the NAD+-dependent histone deacetylases family and involved in several biological processes including apoptosis [28], in the in vitro antitumour effect of DMW [12,29,30]. To further assess the molecular mechanism by which DMW induces cell death in our xenograft model, we investigated the involvement of SIRT3 and SIRT6. Consistent with these previous reports, we found an increase in SIRT3 and SIRT6 protein and mRNA expression in DMW-treated xenograft mice compared to control mice. Although SIRT3 is thought to protect against cell death, it has recently been shown that overexpression of SIRT3 leads to metabolic reprogramming, induction of cell death in CRC, and activation of necroptosis [31]. SIRT3 exerts its effect on tumourigenesis by regulating several genes such as PPAR-α and PPAR-γ [12]. To date, the involvement of PPAR-α as an oncogene has been recognised and PPAR-α has been found to be both overexpressed in CRC and correlated with poor prognosis [32,33]. On the contrary, several lines of research have shown that PPAR-γ plays a crucial role in regulating the growth of various cancers and its expression has been associated with a favourable prognosis for CRC [34]. Consistent with these data, we found that DMW was able to downregulate and upregulate the expression of PPAR-α and PPAR-γ, respectively.

Increasing evidence suggests that CRC tumour progression is regulated by key factors involved in glycolysis [35,36,37]. Lactate dehydrogenase A (LDHA), an enzyme that promotes cancer cell invasion and catalyses the conversion of pyruvate to lactate, is highly elevated in several cancers and is closely associated with cancer progression [18,19,20,21]. Our results show that DMW caused a downregulation of LDHA and consequently a decrease in pyruvate formation, thus supporting the antitumour effect of DMW. All these data provide evidence that DMW induces both apoptosis and necroptosis cell death mechanisms in a xenograft mouse model of CRC.

In conclusion, by revealing whey-induced cell death in a CRC xenograft model, this study paves the way for the potential reuse of this dairy industry byproduct in potential clinical and nutritional strategies against CRC. Considering the ethical debate on the use of chemotherapy in pets, the nutritional strategy against CRC would also be beneficial for fighting cancer in pets.

## 4. Materials and Methods

### 4.1. Milk Whey Preparation

Milk from Italian Mediterranean dairy buffaloes (*Bubalus bubalis*) was obtained from 30 commercial buffalo farms located in the Caserta region of southern Italy. The milk was delactosed by enzymatic hydrolysis with the enzyme lactase (neutral pH and at 25 °C). Subsequently, the whey residue obtained from the ricotta was subjected to reverse osmosis to obtain a concentrated solution of 60% delactosed milk whey (DMW).

### 4.2. Milk Whey Metabolome Profiling

The DMW sample was diluted in methanol 1:100 (*v*/*v*) and then injected directly into the LC-MS system for metabolic profile analysis. This used an Agilent high performance liquid chromatograph (HPLC) 1260 Infinity Series (Agilent Technologies, Santa Clara, CA, USA) equipped with a DAD (diode array detector) system (Agilent Technologies) and coupled to a quadrupole-time of flight (Q-TOF) mass spectrometer (Agilent Technologies) with a Dual ESI source (Agilent Technologies). Chromatographic separation used a Zorbax Extend C-18 column (4.6 × 50 mm, 3.5 µm, Agilent Technologies), maintained at a constant temperature of 30 °C. Elution was carried out at a flow rate of 0.6 mL/min using 0.1% (*v*/*v*) formic acid (FA) in water (H_2_O) as phase A and 0.1% formic acid (FA) in acetonitrile (ACN) as phase B. The gradient was as follows: starting condition 5% B, ramping to 70% B in 4 min, held at 70% B for 1 min, ramping to 80% B in 3 min, and to 100% B in 2 min; finally, lowering to starting condition (5% B) in 5 min and held at 5% B for 1 min as equilibration time. The injection volume was 10 µL. The MS system operated in positive ion mode and MS spectra were recorded in the m/z 50–1700 range as centroid spectra, with a speed of 3.3 spectra/s. The capillary was maintained at 2000 V, fragmentor voltage at 180 V, cone 1 (skimmer 1) at 45 V, Oct RFV at 750 V. Gas flow rate was set at 11 L/min, at 350 °C, and the nebulizer was set at 45 psig. UV spectra were collected by DAD, setting the detection wavelength at 210, 250, and 280 nm. A standard solution was infused by using an isocratic pump (1260 Infinity Series, Agilent Technologies) to perform the real-time lock mass correction. The solution consisted of two reference mass compounds: purine (C_5_H_4_N_4_ at m/z 121.050873, 10 µmol/L) and hexakis (1H, 1H, 3H-tetrafluoropentoxy)-phosphazene (C_18_H_18_O_6_N_3_P_3_F_24_ at m/z 922.09798, 2 µmol/L). Flow rate was set at 0.06 mL/min, while the detection window and the minimum height were set at 1000 ppm and 10,000 counts, respectively, for reference mass correction. HPLC, UV and MS, and parameters were set using Agilent Mass Hunter Data Acquisition Software, rev. B.05.01 (Agilent Technologies).

Chromatographic profiles were evaluated using Mass Hunter Qualitative Analysis, rev B.06.00 (Agilent Technologies) software. Compounds were identified by comparing monoisotopic mass values using a freely available electronic database, the Human Metabolome database (HMDB). Of the molecules detected, only those with a mass error of less than 10 ppm and a sufficient score were reported.

### 4.3. Animals

Mice used in this study were housed in the conventional animal house of the Department of Pharmacy, University of Naples Federico II (Naples, Italy) and kept in cages equipped with additional environmental conditions. For the xenograft model, 6- to 8-week-old female BALB/c nude mice were used (Charles River, Sant’Angelo Lodigiano, Italy), fed ad libitum with sterile mouse chow and kept under pathogen-free conditions in IVC cages. All animals (n = 22) were anaesthetised by inhalation of enflurane before being sacrificed with carbon dioxide. Every effort was made to minimise the number of animals used and their suffering. The mice were blind randomised, and the experimental procedures and protocols were in accordance with national laws and guidelines (Direttiva 2010/63/UE) and approved by the Italian Ministry of Health.

### 4.4. Xenograft Model

The tumour xenograft model of CRC was performed by subcutaneous injection of HCT116 cells (2.5 × 10^6^, 200-μL PBS) into the back of BALB/c nude mice. Ten days after inoculation (once the tumours had reached a size of 150–250 mm^3^), the mice were randomly assigned to control and treated groups, and treatments were initiated. Tumour size was measured daily with a digital calliper, and tumour volume was calculated using the modified ellipsoid volume formula (volume = π/6 × length × width^2^). DMW was given orally, at the dose of 0.2 mL/mouse, daily for the duration of the experiment. Eleven days after the start of treatment, the mice were sacrificed, and the tumours were removed, measured by digital calliper, and fixed in formalin or stored at −80 °C for subsequent analyses [38].

### 4.5. Histological Examination

Tissue samples were fixed with 10% formalin and embedded in paraffin. Sections were cut at 4 µm, stained with haematoxylin and eosin (H-E), and viewed on microscope (Nikon Eclipse E-400, Tokyo, Japan) by two independent observers for histopathological examination, which confirmed the diagnosis of colon adenocarcinoma. The extent of tumour necrosis was assessed semiquantitatively, evaluated at low magnification (×40) according to previously published criteria [39,40]**,** and recorded as mild (score 1; ≤10% of tumour area), moderate (score 2; 10–30% of tumour area), and severe (score 3; ≥30% of tumour area).

### 4.6. RNA Isolation and Detection

Total RNA was purified for each sample using Trizol reagents (Invitrogen, Life Technologies, Carlsbad, CA, USA). The protocol was performed according to the manufacturer’s instructions. The final elution step was repeated, and the purified total RNA was then stored at −80 °C. The amount of total RNA samples was determined using the Thermo Scientific™ NanoDrop™ 2000 spectrophotometer. The level of degradation was analysed by agarose electrophoresis. Samples with distinct 28S and 18S ribosomal RNA bands and band intensities of 28S:18S close to two were selected for this study.

### 4.7. cDNA Synthesis

First-strand complementary DNA (cDNA) was synthesised with 1 μg total RNA using the QuantiTect^®^ Reverse Transcription Kit (Qiagen, Germany) following the manufacturer’s recommendations. Genomic DNA contaminating the RNA samples was removed using 2 μL gDNA wipeout buffer, 1 μg template RNA and RNase-free water to bring the final volume to 14 μL at 42 °C for 2 min. Reverse transcription was carried out using 1 μL Quantiscript reverse transcriptase, 4 μL Quantiscript RT buffer containing Mg^2+^ and dNTPs, 1 μL RT primer mix, and 14 μL template RNA in a total of 20 μL reaction at 42 °C for 15 min and 95 °C for 3 min. All steps were performed on ice. cDNA samples were stored at −20 °C for later quantitative real-time PCR analysis.

### 4.8. Quantitative Real-Time PCR Analysis

Quantitative real-time PCR reactions were performed using the QuantiFast^®^ SYBR^®^ Green PCR Kit (Qiagen, Germany) on the BIORAD CFX Manager Machine. All primers were designed by the Primer-BLAST software and synthesised by Eurofins Genomics (Ebersberg, Germany). Samples were prepared according to the manufacturer’s instructions. A total reaction volume of 20 μL contained 10 μL master mix, 2 μL primer, and 8 μL diluted cDNA (1:30). Samples were then centrifuged at 1000× *g* at 4 °C for 1 min. All cDNA samples were amplified under the following conditions: 95 °C for 5 min, 40 cycles at 95 °C for 10 s, and 60 °C for 30 s. Primers used in this study were: PARP Fw: GCAGAGTATGCCAAGTCCAACAG Rv: ATCCACCTCATCGCCTTTTC, CASP3 Fw: GCAGCAAACCTCAGGGAAAC Rv: TGTCGGCATACTGTTTCAGCA, MLKL Fw: GTGGGAAAGAAGGTGGAAGAG Rv: GCCAAGGGTGATAATATGCTTC, β-ACTIN Fw: CATGTACGTTGCTATCCAGGC Rv: CTCCTTAATGTCACGCACGAT. Data were then quantified using the comparative cycle threshold (CT) method and expression levels were presented as 2^−ΔCT^ normalised to the β-actin housekeeping gene.

### 4.9. Lipid Peroxidation Detection

The level of lipid peroxidation was determined by measuring the cellular content of malondialdehyde (MDA) using the colorimetric lipid peroxidation assay kit (ab233471, Abcam, Cambridge, UK). According to the manufacturer’s instructions, a solution containing 20 mM Na phosphate buffer at pH 3 and 0.5% TritonX-100 was added to the tissue extracts to avoid non-specific aldehydes that could interfere with absorbance measurements. MDA Colour reagent solution (10 μL) was added to each well and the reaction mixture incubated at room temperature (RT) for 30 min. The reaction solution (40 μL) was also added followed by a second incubation at RT for 1 h. Absorbance was measured at 695 nm using a microplate reader (model 680, Bio-Rad, Hercules, CA, USA) and total MDA content was calculated by comparing the absorbance of each sample to the standard MDA curve (0–200 μM) and normalising to protein content.

### 4.10. Measurement of Pyruvate Levels

The measurement of pyruvate levels in tissue extracts was performed with Pyruvate Assay Kit (ab65342, Abcam), according to manufacturer’s protocol. Deproteinisation was carried out using a 10 kDa spin filter (Amicon Ultra-0.5 mL Centrifugal Filter) with centrifugation at 14,000× *g* for 10 min at 4 °C. Then, reaction mix (50 μL) was added to pyruvate standard and samples (50 μL) and plate were incubated for 30 min at RT in the dark. The absorbance was measured at 570 nm with a microplate reader (model 680, Bio-Rad) and pyruvate content interpolated from the standard curve.

### 4.11. SIRT3 and SIRT6 Enzyme Activity

SIRT3 and SIRT6 fluorometric activity assay kits (ab156067 and ab156068, Abcam) were performed according to the manufacturer’s instructions. Briefly, Fluoro-substrate peptide, NAD, developer, and mouse tissue extracts (5 μL) were added to each well of the plate by mixing thoroughly at RT. After 1 h incubation, the reaction was stopped with 20 μL of stop solution to each well, and SIRT3 and SIRT6 activity resulted in fluorescence intensity, measured in a multiplate reader (Infinite 2000, Tecan, Männedorf, Switzerland) capable of excitation at a wavelength in the range of 340–360 or 480–500 nm and detection of emitted light in the range of 440–460 or 520–540 nm.

### 4.12. Immunoblotting Analysis

Proteins from tissue extract (20–50 µg) were resolved by sodium dodecyl sulfate-polyacrylamide gel electrophoresis (SDS-PAGE) and transferred to nitrocellulose membranes (Bio-Rad). Blots were blocked for 1 h at RT in 1× TBS 1% casein blocker (1610782, Bio-Rad) under a gentle shaker. Membranes were then incubated overnight at 4 °C with specific primary antibodies: anti-SIRT3 (1:2000, PA5-86035, Invitrogen, Waltham, MA, USA); anti-SIRT6 (1:1000, ab191385, Abcam); antiperoxisome proliferator-activated receptor α (PPAR-α, 1:1000, E-AB-32646, Elabscience Biotechnology Inc., Houston, TX, USA); anti-PPAR-γ (1:1000, orb69095, Biorbyt, Cambridge, UK); antilactate dehydrogenase A (LDHA, 1:1000, PA5-27406, ThermoFisher Scientific, Waltham, MA, USA); antimixed lineage domain-like protein (MLKL, 1:1000, ab196436, Abcam), antireceptor interacting protein 1 (RIPK1/RIP1, 1:1000, SAB3500420, Sigma-Aldrich, St. Louis, MO, USA), anti-RIPK3/RIP3 (1:1000, NBP1-77299, Novus Biologicals, Littleton, CO, USA), antinuclear factor kappa B (NF-κB, 1:1000, C22B4, Cell Signaling Technology, Danvers, MA, USA); cleaved PARP (#5625, Cell Signaling Technology, 1:1000,), cleaved caspase-3 (#9664 Cell Signaling Technology, 1:1000), Phospho-p44/42 MAPK Erk1/2 Thr202/Tyr204 (#4370, Cell Signaling Technology,1:1000), p44/42 MAPK Erk1/2 (#4695S, Cell Signaling Technology Inc., USA,1:1000), Phospho-Histone H3 (Ser10) (#9701, Cell Signaling Technology Inc., USA,1:1000), c-Myc (#18583, Cell Signaling Technology Inc., USA,1:1000), anti-α-tubulin (1:5000, E-AB-20036, Elabscience Biotechnology Inc.); anti-actin (1:3000, ab179467, Abcam), and anti-glyceraldehyde-3-phosphate dehydrogenase (GAPDH, 1:2000, ab9485, Abcam). After 1 h incubation with HRP-conjugated secondary antibodies (NC GxMu-003-DHRPX and GtxRb-003-DHRPX, ImmunoReagents Inc., Raleigh, NC, USA), the immunocomplexes were visualized, with Excellent chemiluminescent substrate kit (E-IR-R301, Elabscience Biotechnology Inc.), using the ChemiDoc Imaging System with Image Lab 6.0.1 software (Bio-Rad Laboratories). After background subtraction, the densities of immunoreactive bands were obtained by ImageJ 1.52n software (National Institutes of Health) and expressed as arbitrary units (AU). For Phospho-p44/42 MAPK Erk1/2 Thr202/Tyr204, p44/42 MAPK Erk1/2, Phospho-Histone H3 (Ser10), c-Myc, cleaved PARP, and cleaved caspase 3, immunoreactive bands were detected on X-ray films using an enhanced chemiluminescent kit and quantified by densitometry using ImageJ software.

### 4.13. Confocal Laser Scanning Microscopy

Immunofluorescence analysis was performed on deparaffinised sections from mice. Samples were placed in antigen retrieval buffer (10 mM sodium citrate, 0.05% Tween 20, pH 6.0) and heated in the microwave for 20 min. To reduce background fluorescence, the slides were washed twice in phosphate-buffered saline (PBS) and incubated for 30 min in Tris-buffered saline (TBS) containing 50 mM ammonium chloride. Permeabilisation with PBS containing 0.2% Triton X-100 was carried out for 10 min at RT. The sections were blocked with foetal bovine serum (FBS) and saponin (0.1 g/mL) for 1 h at RT, and then, incubated with primary antibodies: anti-MLKL antibody (1:300), anti-SIRT6 (1:500), anti-SIRT3 (1:300), anti-caspase-3 (1:300, orb10237, Biorbyt), and anti-PARP (1:500, ab194586, Abcam), before 1 h incubation at RT with secondary antibodies labelled with Alexa Fluor 633. For all slides, cellular architecture was marked by Phalloidin 488 (1:1000, ab17675, Abcam) and nuclei stained with 4,6-diamidino-2-phenylindole (DAPI, 5 μg/mL) for 7 min. Sections were quenched for autofluorescence using the Vector TrueVIEW Autofluorescence Quenching Kit (VEC-SP-8500, Vector Laboratories, Newark, CA, USA) and mounted in Vectashield Mounting Medium (H-1700, Vector Laboratories). Slides were imaged using a Zeiss LSM 700 confocal microscope (Zeiss, Oberkochen, Germany) with a plan apochromat ×63 (NA1.4) oil immersion objective and fluorescence intensity values, reported as arbitrary fluorescence units (AFU), evaluated by ImageJ 1.52n software (National Institutes of Health).

### 4.14. Assessment of Cytokine Content

Cytokine (IFN-γ and TNF-α) levels in mouse extracts were determined by ELISA assays (ab100690, IFN-γ mouse ELISA kit, Abcam; MBS825075, mouse TNF-α ELISA kit, MyBioSource, San Diego, CA, USA), according to the manufacturer’s instructions. Standards and samples (100 μL) were incubated overnight at 4 °C with gentle shaking to allow for cytokines binding to the wells, by the immobilised antibody, of precoated 96-well plate. The wells were washed 3 times and biotinylated antimouse IFN-γ and TNF-α antibodies (100 μL) were added and the plate was incubated at 37 °C for an additional hour. After washing away unbound antibody, HRP-conjugated streptavidin (100 μL) was added to each well and, after 45 min at 37 °C and 4 washes, TMB substrate solution (100 μL) was included for 30 min to develop colour, in proportion to the amount of cytokines. The addition of stop solution (100 μL) allowed a colour change from blue to yellow and the colorimetric intensity was measured at 450 nm using a microplate reader (model 680, Bio-Rad). The content of cytokines in the samples was determined by plotting the absorbance values against the concentrations of the individual standard curves.

### 4.15. Statistical Analysis

All data are expressed as mean ± SEM. Statistical analysis was performed using GraphPad Prism 9 software (La Jolla, San Diego, CA, USA). Comparisons between tissue samples from vehicle- and DMW-treated mice were performed using the two-tailed unpaired Student’s *t*-test. A *p*-value < 0.05 was considered significant.

## 5. Patents

Patent n. 102021000024911; deposition date, 29 September 2021.

## Figures and Tables

**Table 1:** Compounds identified in delactosed milk whey from Italian Mediterranean dairy buffaloes (*Bubalus bubalis*) by HPLC-MS analysis.

RT (min)	Experimental m/z [M-H]^+^ (Da)	Theoretical Mass (Da)	Mass Error (ppm)	Molecular Formula	Putatively Identified Compound	Metabolite Class
0.698	198.9395	197.93501	1.00449	C_4_H_7_BrO_2_S	3-Bromosulfolane	Triterpenoid
0.758	365.1047	364.094688	1.010012	C_21_H_16_ O_6_	Gerberinol	4-Hydroxycoumarin
0.81	203.0521	202.047738	1.004362	C_8_H_10_O_6_	Ethyl aconitate	Trycarboxylic acid
0.821	162.1119	161.105193	1.006707	C_7_H_15_NO_3_	L-Carnitine	Amino acid
0.832	160.1325	159.125929	1.006571	C_8_H_17_NO_2_	Δ-Valerobetaine	Straight chain fatty acids
0.847	204.1226	203.115758	1.006842	C_9_H_17_NO_4_	Acetyl-L-carnitine	Amino acid
0.873	381.0778	380.077718	1.000082	C_14_H_20_O_10_S	4-Methoxybenzyl O-(2-sulfoglucoside)	O-glycoside
0.911	232.1535	231.147058	1.006442	C_11_H_21_NO_4_	Butyryl-L-carnitine	Amino acid
5.951	230.2481	229.240565	1.007535	C_14_H_31_NO	Xestoaminol C	1.2-Aminoalcohol
9.638	338.3404	337.334465	1.005935	C_22_H_43_NO	Erucamide	Fatty acid amide

## Data Availability

Not applicable.

## Supplementary Materials

The following supporting information can be downloaded at: https://www.mdpi.com/article/10.3390/ijms23158464/s1.
