# Supplementary material for: Buffalo Milk Whey Activates Necroptosis and Apoptosis in a Xenograft Model of Colorectal Cancer
Source: Int J Mol Sci. 2022 Jul 30;23(15):8464. doi: 10.3390/ijms23158464 (PMC9368892; doi:10.3390/ijms23158464)
Supplement: Supplementary file 1 [file ijms-23-08464-s001.zip › ijms-1830636-supplementary.pdf]

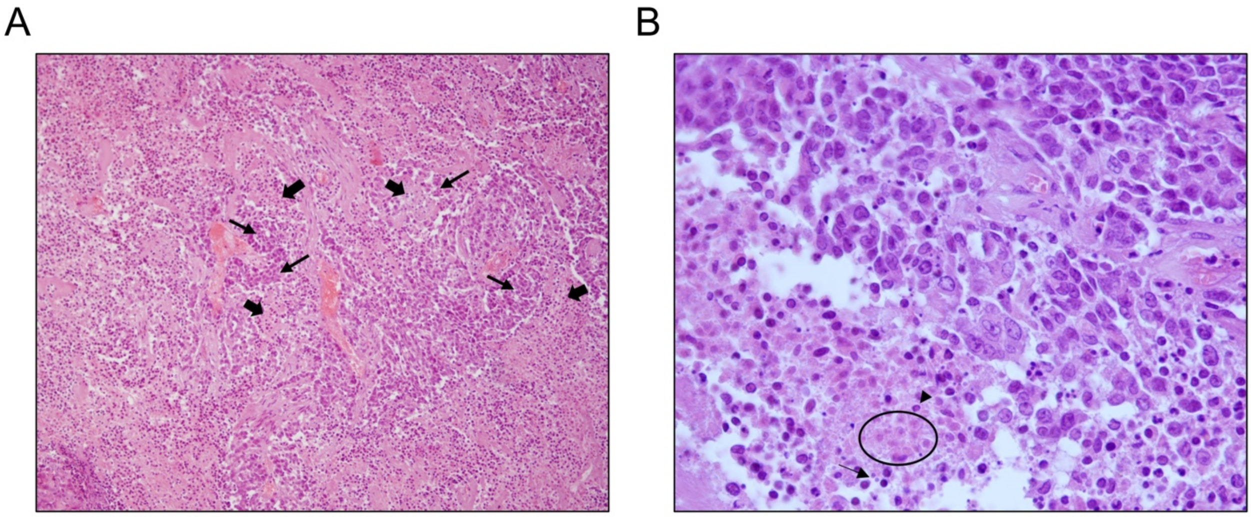

Figure S1. (A) Xenograft tissue from DMW-treated mice showed necrosis (thick arrows) invading small neoplastic areas and single neoplastic cells (thin arrows) **(B)** Xenograft tissue from DMW-treated mice showed neoplastic cells with clear signs of pyknosis (arrowhead), karyorrhexis (arrow) and karyolysis (ghost cells) (circle). (H-E; original magnification ×10).

Supplementary Table S1. Necrosis entity in xenograft tumour tissue from DMW-treated or untreated mice.

NUMBER OF SAMPLES	NECROSIS ENTITY
**Control**	
**7**	MILD
**3**	MODERATE
**1**	SEVERE
**DMW**	
**3**	MILD
**3**	MODERATE
**5**	SEVERE
